# Olive Leaf Addition Increases Olive Oil Nutraceutical Properties

**DOI:** 10.3390/molecules24030545

**Published:** 2019-02-02

**Authors:** Imen Tarchoune, Cristina Sgherri, Jamel Eddouzi, Angela Zinnai, Mike Frank Quartacci, Mokhtar Zarrouk

**Affiliations:** 1 Laboratoire de Biotechnologie d’Olivier, Centre de Biotechnologie de Borj-Cedria, B.P. 901, 2050 Hammam-Lif, Tunisia; imen_tarchoune@yahoo.fr (I.T.); zarrouk.mokhtar@gmail.com (M.Z.); 2 Department of Agriculture, Food and Environment (DAFE), Via del Borghetto 80, 56124 Pisa, Italy; angela.zinnai@unipi.it; 3 Laboratoire de Biotechnologie Appliquée à l’Agriculture, INRA Tunis, Université de Carthage, El Menzah, 1004 Tunis, Tunisia; douzi.jamel@gmail.com; 4 Interdepartmental Research Centre “Nutraceuticals and Food for Health”, University of Pisa, Via del Borghetto 80, 56124 Pisa, Italy

**Keywords:** extra virgin olive oil, leaf addition, Tunisian varieties, phenolics, tocopherols, antioxidant activity

## Abstract

The aim of the present research was to study the effects of olive leaf addition (0 and 3%) on the major antioxidants and the antioxidant activity of Neb Jmel and Oueslati olive oils. Olives and leaves of the two Tunisian varieties were harvested during the 2016/2017 crop season. Both leaves and oils were characterised for their concentrations in phenolics, tocopherols and antioxidant power. Other parameters such as free acidity, peroxide value, chlorophyll and carotenoid concentrations were also taken into consideration. Compared to Oueslati, the Neb Jmel oil showed a lower free acidity (50%) and peroxide value (5.6-fold), and higher chlorophyll (1.6-fold), total phenolics (1.3-fold), flavonoid (3-fold) and oleuropein derivative (1.5-fold) concentrations, in addition to an increased antioxidant activity (1.6-fold). Leaf addition promoted a significant increment in total chlorophyll, α-tocopherol and phenolics in both varieties, above all in Oueslati oil, due to a higher abundance of bioactive constituents in the corresponding leaves. In particular, chlorophyll and carotenoid concentrations reached values twice higher than in Neb Jmel leaves, and flavonoids and oleouperin derivatives were three-fold higher. This prevented the oxidation and the formation of peroxides, reducing the peroxide value of the fortified oil to the half. The results provide evidence on the performance of the Tunisian Neb Jmel and Oueslati varieties, showing that their oils present a chemical profile corresponding to the extra virgin olive oil category and that, after leaf addition, their nutritional value was improved.

## 1. Introduction

In the Mediterranean area, the olive tree is so economically important that oliviculture is one of the most widespread agricultural activities. It is estimated that about 8 million ha are cultivated with olive trees [1,2]. In particular, in Tunisia, an area of 1.7 million ha is covered by olive trees, which results in the production of more than 4% of the global olive oil amount. Due to the 170,000 tons produced per year, Tunisia is the fourth largest producer and exporter of olive oil in the world [3].

The nutritional and health-promoting effects of olives and olive oils are more and more recognized [4]. Indeed, it is well known that olive oil is a potential antioxidant [5,6,7] showing anti-inflammatory [6,8,9], cardioprotective [6,10], anticancer [6,11], antidiabetic [12] and neuroprotective effects [13].

The importance of olive oil is related to its high amounts of monounsaturated fatty acids and to the presence of low-represented components such as α-tocopherol, phenolics, chlorophyll and carotenoids. Phenols are among the most important nutraceutical compounds because of their nutritional and sensorial characteristics [14]. Phenolic compounds and tocopherols play a protective role against oxidative stress [15] and are able to extend the extra virgin olive oil shelf-life due to their antioxidative properties [16]. Olive oil colour is determined by its pigment composition and concentration, especially with reference to chlorophyll. In the dark, this pigment is also endowed with antioxidant activity, even if under light conditions it can act as a prooxidant, reacting with triplet oxygen to form the excited-state singlet oxygen [17]. Oxidation is the most important process that causes quality deterioration of olive oil because of its high concentration of unsaturated fatty acids. Consequently, synthetic antioxidants have been used as food additives to improve oil stability. As many studies suggest that the use of synthetic antioxidants may lead to health risks [18], considerable attention has been recently focused on plant phenolics due to their healthful and nutritional effects [19]. Recent papers have provided evidence that olive leaves have a high antioxidant activity, originated by the presence of phenolics, thus exhibiting strong preventive effects against oxidation [18,20,21]. Olive leaves are a by-product of olive cultivation and can be easily obtained either from pruning or olive oil industry as a waste product [1].

The present research was designed to verify a possible improvement of the nutritional value of two Tunisian olive oils by adding olive leaves, as a source of natural antioxidants, during oil extraction.

## 2. Results and Discussion

### 2.1. Quality Parameters

According to the definitions and standards established by the International Olive Oil Council [22], the classification of virgin olive oil into different categories depends on its chemical, physical and sensory parameters, among which the degree of acidity and the peroxide value (Table 1) are the most used. In this study, the differences observed between the two oils could be due, besides variety, to the different geographical location of the plants in terms of climate and soil composition.

### 2.2. Free Acidity

Oil acidity is a simple and effective parameter to evaluate and classify a commercial-grade olive oil [23]. The extraction of olive oil from fresh undamaged fruits carried out following a correct crushing procedure gives oils with very low acidity [23]. However, during extraction and storage the olive oil can be altered by the release of free fatty acids as a consequence of the hydrolysis of triglycerides, thus increasing the free acidity.

The acidity values of Neb Jmel and Oueslati olive oils, extracted without (control) and with the addition of 3% olive leaves, are reported in Table 1. The Neb Jmel oils showed very low values (0.6%). The addition of leaves during oil extraction did not affect the free acidity in this variety. Regarding Oueslati, acidity of oils (1%) was higher in comparison with the Neb Jmel ones. However, the addition of leaves influenced Oueslati oil quality, decreasing free acidity to 0.6%. Our results agree with those reported by Ben Mansour et al. [24] for the Neb Jmel variety, whereas the free acidity of Oueslati oils was higher in comparison with the findings of Ouni et al. [25] on the same variety. This behaviour could be related to the use of olives at an advanced stage of maturation. Indeed, a late harvest of olives may alter oil acidity by increasing the lipolytic enzyme activities [26]. However, in our case the addition of olive leaves was responsible for the decrease of free acidity likely due to the presence of antioxidant compounds. Consequently, fortified Oueslati oils could still be classified as extra virgin olive oils as the free acidity value was lower than 0.8%.

### 2.3. Peroxide Value

Peroxides are intermediate products of oil oxidation which originate a complex mixture of volatile compounds such as aldehydes, ketones, hydrocarbons, alcohols and esters. These compounds are responsible for the alteration of the organoleptic characteristics [27], dramatically reducing oil shelf-life as well as consumer acceptance. Furthermore, also light and high temperatures are well-known factors generally promoting peroxide formation [28].

Table 1 shows that the peroxide value of Neb Jmel oils was well below the established limit (<20 milliequivalents (meq) O_2_/kg) for all the categories of olive oil [22]. In contrast, a high peroxide value was observed in the Oueslati control oil (34 meq O_2_/kg), so that it could no longer be classified as an extra virgin olive oil. However, the addition of leaves during oil extraction and processing prevented the oxidation and the formation of peroxides, reducing the peroxide value to half. Contrary to our results, Malheiro et al. [17] reported that leaf addition increased the peroxide value. Such discrepancy could be explained with differences in the relative presence of additional antioxidants and of leaf residues. These constituents, through gas exchanges occurring during the respiration process, may have increased the availability of oxygen, thus inducing peroxidation.

### 2.4. Chlorophylls and Carotenoids

Chlorophylls and carotenoids play important roles in olive oils. They interfere with the oxidative stability, acting as antioxidants in the dark or as prooxidants when exposed to light [29]. Furthermore, these compounds are responsible for the yellow-green pigmentation of olive oils, increasing consumer acceptability. Chlorophyll and carotenoid concentrations of olive oils and leaves are reported in Table 1 and Table 2, respectively.

The Neb Jmel oil showed a higher chlorophyll value (Table 1) in comparison with Oueslati. However, Oueslati leaves (Table 2) displayed the highest chlorophyll (829.29 μg/g) and carotenoid (44.3 μg/g) concentrations, highlighting the role that the addition of chlorophyll could have had in the oxidative stability of the olive oil. This can further explain the difference with the results reported by Malheiro et al. [17] concerning the peroxide value, as in their study olive leaf addition did not affect the chlorophyll content.

In the present work, the addition of leaves (3%) to Neb Jmel and Oueslati oils enhanced chlorophyll and carotenoid concentrations. In fact, chlorophyll reached the same level in both fortified oils, even if Oueslati oil was the one that showed the highest carotenoid concentration. Therefore, significant effects of leaf addition on oil pigments were observed in both varieties (Table 1 and Table 2). The addition of leaves also turned the olive oils greener, this visual observation being clear in both oils, and was very likely associated with the increased pigment concentration. The increase in chlorophyll concentrations makes the fortified oils interesting from a nutritional point of view due to the antioxidant activity of chlorophyll and its potential to exert chemopreventive actions against carcinogens [30].

### 2.5. Total Phenolics

Phenolics are important components for olive oil quality and organoleptic characteristics. Moreover, they are very effective antioxidants playing an important role in human diet and health [3]. Current evidences strongly support the contribution of phenols to the prevention of cancer, cardiovascular and neurodegenerative diseases. The shelf-life of an oil is also correlated with its natural antioxidant amount [16]. Indeed, phenolics delay the oxidative degradation process, thus extending the shelf-life of the product [3,15].

**Figure 1:**
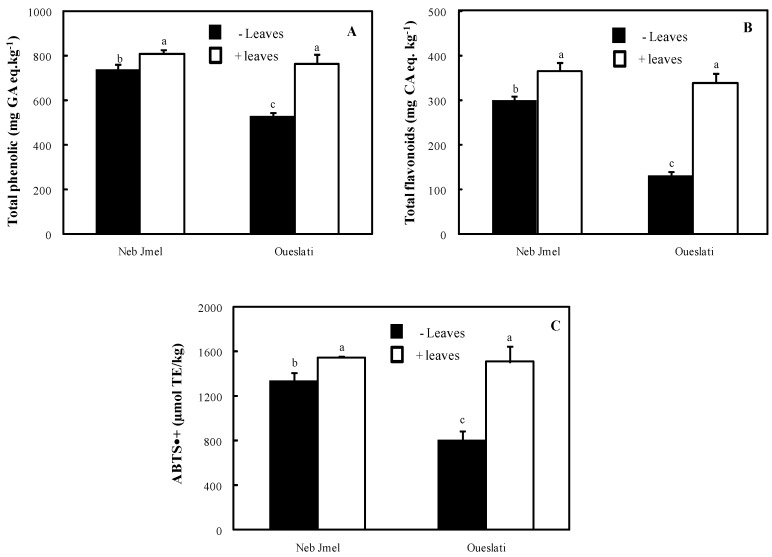
Total phenol (**A**) and total flavonoid (**B**) contents and ABTS^•+^ scavenging activity (**C**) of Neb Jmel and Oueslati olive oils extracted with and without addition of olive leaves (3%). Data are means of three independent experiments ± SE (*n* = 3). Means followed by different letters are significantly different at *p* ≤ 0.05 as determined by Duncan’s multiple range test.

Figure 1 shows that total phenolic concentration of the Neb Jmel oil (736 mg GA eq/kg oil) was significantly higher than that of Oueslati (528 mg GA eq/kg oil). In the literature, it was reported that the total phenolic concentration of Neb Jmel olive oils varies from 562 to 1167 mg GA eq/kg oil [24], whereas for Oueslati it changes from 100 to 859 mg GA eq/kg oil [25,31]. The effect of the geographic location on phenols can be evidenced from the different behaviour showed by each variety [24,25,31]. Indeed, Neb Jmel oil (from the north of Tunisia) showed a higher phenol concentration than Oueslati (from central Tunisia). The present data confirm previous findings on the effect of the variety on phenol concentration in oils [24,25,31].

Oueslati leaves showed a higher phenolic concentration than Neb Jmel ones (Table 2). It is worth noting that leaf addition, increasing total phenolic concentration by 44 and 10% in Oueslati and Neb Jmel oils, respectively, (Figure 1), determined the same mean value in the fortified products. Similar findings were also confirmed by other studies [32,33].

### 2.6. Total Flavonoids

Flavonoids are plant secondary metabolites with different phenolic structures. These compounds are used mostly to generate pigments which play an important role in the colours of plants. During the past decade, many studies have reported their beneficial effects on human health [34,35]. Indeed, flavonoids display important anti-inflammatory, antiallergic and anticancer activities as well as antiviral properties [34,35]. In this study, total flavonoid concentration was determined in both the oils and leaves of the two Tunisian olive cultivars Neb Jmel and Oueslati (Table 2, Figure 1). The results point out that Oueslati leaves showed a concentration of total flavonoids two-fold higher than Neb Jmel. However, in Oueslati oil the total flavonoid content was about half than that found in Neb Jmel one (Figure 1). The effect of leaf addition during oil extraction on these compounds was remarkable. Our results showed an increase in total flavonoids by 22% in Neb Jmel oils and by 160% in Oueslati, thus determining a not significant difference between the two fortified oils. This was likely due to the highest level of total flavonoids of Oueslati leaves. According to the present research, the findings of Ebrahimi et al. [36] reported values ranging from 156 to 361 mg rutin eq/kg for refined and crude olive oils, respectively.

### 2.7. ABTS^•+^ Scavenging Activity

ABTS^•+^ scavenging activity of the Neb Jmel oil was 66% higher than that of Oueslati (Figure 1). In contrast, Oueslati leaves showed a higher free-radical scavenging activity compared to the Neb Jmel variety (Table 2). Following 3% leaf addition, the antioxidant capacity was increased by 15% and 87% in Neb Jmel and Oueslati oils, respectively. This wide change was probably due to the increases in chlorophyll, carotenoid, total phenolic and flavonoid concentrations. Indeed, some authors found a good correlation between the total phenolic amount and the radical scavenging power [37,38].

### 2.8. Phenolic Compounds

The analysis of the phenolic profile of olive leaf extracts and oils from the two varieties is reported in Table 3 and Table 4, respectively. Thirteen phenolic compounds, among which phenolic acids, phenolic alcohols and secoiridoids, were identified and quantified. As regards olive leaf extracts, oleuropein derivatives were the most represented, followed by phenolic acids and phenolic alcohols (Table 3).

With minor changes compared to leaves, oleuropein derivatives were still the most abundant compounds in oils, followed by phenolic alcohols and phenolic acids (Table 4). In particular, Oueslati leaves showed a three-fold higher content in oleuropein derivatives than Neb Jmel ones, whereas Neb Jmel oil exhibited the highest concentration of these compounds. With leaf addition, oleuropein derivative concentration increased by 9 and 48% in Neb Jmel and Oueslati oils, respectively, confirming that olive leaves are a source of oleuropein derivatives [39]. Consistent with other reports, we found that oleuropein derivative amounts changed depending on the variety.

During the past few years, the biological activities of olive oil phenolics, namely oleuropein derivatives, have been thoroughly investigated. Several studies have attempted to elucidate the performance of oleuropein derivatives as antioxidant compounds. As the excessive presence of reactive oxygen species has been suggested to participate in the aetiology of several diseases [40], the focus on powerful antioxidants able to counteract the free-radical attack has become increasingly important. The antioxidant actions of oleuropein have been mostly assigned to its free-radical scavenging activity. Considering all together, the addition of olive leaves during oil extraction process suggests that it could be a means for improving oil quality.

Although olive fruits are rich in secoiridoids, hydroxytyrosol and tyrosol represent the two most important phenolic alcohols of both olive leaves [41] and oils [42]. Leaf composition of the Oueslati variety was characterised by a four-fold higher amount of hydroxytyrosol and tyrosol compared to Neb Jmel (Table 3). However, the oils, and likely the fruits, were mostly endowed with these phenolic alcohols in the Neb Jmel variety, showing concentrations of hydroxytyrosol and tyrosol of 3.57 and 17.97 mg/kg, respectively (Table 4). Obviously, the difference in the amounts of phenolic alcohols (hydroxytyrosol and tyrosol) depends not only on the variety, but also on the organ—leaf or fruit—considered. Our findings are in agreement with previous studies on phenolics in olive oil [17,21]. Oueslati oil, after 3% leaf addition, did not show any significant change in the hydroxytyrosol and tyrosol contents in comparison with the control (Table 4). These results suggest that a very high content of phenolics and related compounds could have ended up in olive mill wastewater. Indeed, many investigations clearly showed the occurrence of a high content of phenolic compounds in olive mill wastewater [43,44,45,46]. The hydrophilic character of polyphenols was likely responsible for the solubilisation of the most part of phenols into the water phase during oil extraction.

Phenolic acids found at considerable concentrations in Neb Jmel and Oueslati leaves (Table 3) were represented by gallic, protocatechuic, *p*-hydroxybenzoic, chlorogenic, vanillic, caffeic, syringic, vanillin, *p*-coumaric and ferulic acids. In the oils of the two varieties, the identified phenolic acids did not overcome the value of 2 mg/kg (Table 4), which agrees with the results reported by Kelebek et al. [47]. In both olive varieties, no significant effect of leaf addition was registered.

### 2.9. Tocopherols

**Figure 2:**
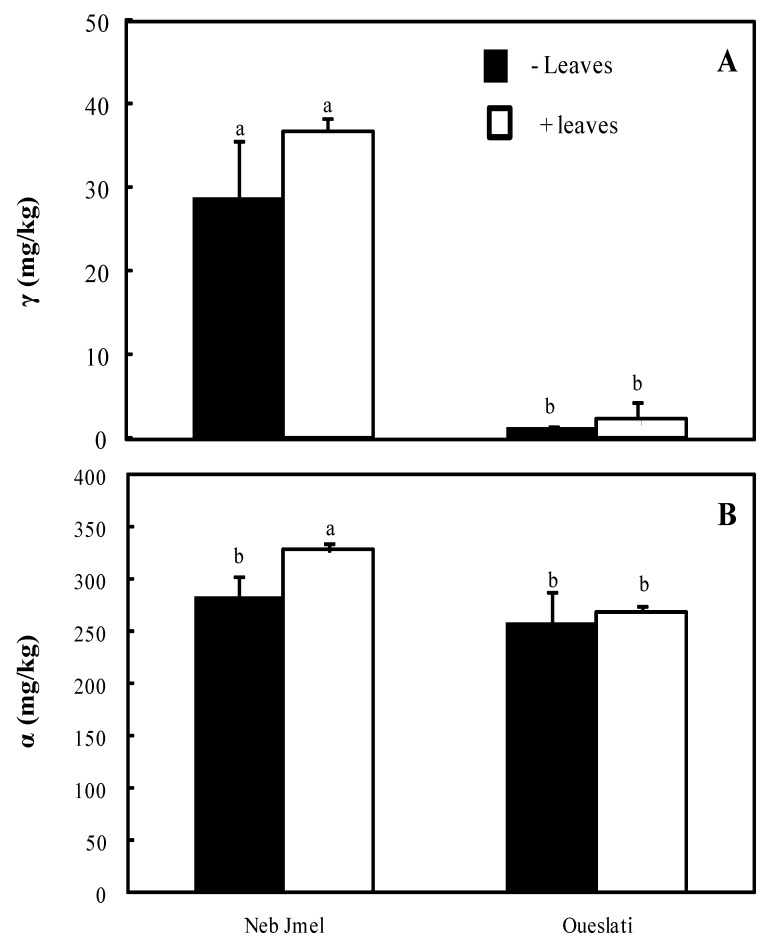
γ-Tocopherol (**A**) and α-tocopherol (**B**) content of Neb Jmel and Oueslati olive oils extracted with and without addition of olive leaves (3%). Data are means of three independent experiments ± SE (*n* = 3). Means followed by different letters are significantly different at *p* ≤ 0.05 as determined by Duncan’s multiple range test.

Tocopherols occur in vegetable oils, playing an important role during oxidative processes. Two identified compounds (α- and γ-tocopherol) were quantified in this study. The quantitative profile of α- and γ-tocopherol is shown in Table 2 and Figure 2.

Tocopherol concentration in olive leaves is reported in Table 2. α-Tocopherol was present at higher amounts in Neb Jmel leaves (82.37 μg/g DW) in comparison with Oueslati, which showed an eight-fold lower amount (10.12 μg/g DW); γ-tocopherol was found in trace amounts in the leaves of both varieties.

In control oils (Figure 2), the α-isomer reached values of 257.8 and 283.6 mg/kg in Oueslati and Neb Jmal, respectively, not showing any significant difference between the two cultivars. Following leaf addition, α-tocopherol concentration showed a slight increase in the Neb Jmel oil, where it reached the value of 328.08 mg/kg. This result could be related to the higher presence of this compound in the corresponding leaves (Table 2). In contrast, the lower amount of α-tocopherol detected in Oueslati leaves (10.12 μg/g) did not determine any improvement in the oil following leaf addition (Figure 2). According to Malheiro et al. [17], the amount of α-tocopherol in oils was not significantly influenced when leaf addition was less than 5%.

Concerning γ-tocopherol (Figure 2), the amount was very low compared to the α-isomer (1.17–28.73 mg/kg), being it more represented in the Neb Jmel oil. Leaf addition (3%) did not significantly affect γ-tocopherol concentrations due to the fact that in the leaves of both varieties this isomer was detected in trace amounts (Table 2).

Consistent with previous reports [48], we found that olive leaves can be used as an alternative source to improve the chemical composition of olive oils, mainly the α-tocopherol concentration. Likewise, the present results agree with previous studies on the influence of the cultivar on α-tocopherol concentration. Franco et al. [49] reported very high levels of α-tocopherol in seven varieties of Spanish oils (217–345 mg/kg). In contrast, in the Portuguese olive oil studied by Cunha et al. [50], values ranging from 93 to 260 mg/kg were found. Similar values to those reported in the present experiment were found in some studies performed on different Tunisian oils [51,52,53].

Antioxidants, such as vitamin E (tocopherols), may prevent the detrimental effects of free radicals. In the Mediterranean diet, olive oil substantially contributes to the daily intake of these antioxidants. The health benefits of vitamin E are evidenced by the fact that the ingestion of fresh fruits and vegetables is inversely related to the extent of some cancers as well as to plasma lipid peroxidation [54]. It should be highlighted that the two vitamin E isoforms have different health-related properties. In fact, γ-tocopherol is the less powerful antioxidant, although being capable of trapping peroxynitrites. For this reason, γ-tocopherol has been acknowledged as the “other” vitamin E important for human health [54].

## 3. Materials and Methods

### 3.1. Olive Leaves and Fruit Sampling

The olives of two varieties were collected from different regions: Oueslati, in the centre (Khit El Oued), and Neb Jmel, in the north (Borj El Amri) of Tunisia. The harvest was performed at the same stage of maturity, considering a colour maturity index of about three. The maturity index was evaluated taking into consideration the changes in skin and pulp colours. Samples of 100 fruits were taken randomly and classified into eight groups or categories: green intense (category 0), yellow or yellowish green (category 1), green with reddish spots (category 2), reddish or light violet (category 3), black with white pulp (category 4), black with ˂50% purple flesh (category 5), black with ≥50% purple flesh (category 6) and black with 100% purple flesh (category 7). The maturity index was calculated as A × 0 + B × 1 + C × 2 + D × 3 + E × 4 + F × 5 + G × 6 + H × 7/100, where A, B, C, D, E, F, G and H are the number of fruits in each class.

The olives were picked by hand from three trees during the 2016/2017 crop season (November). Olive leaves were also collected from the same trees at harvest. The following percentages of olive leaves were added to fruits (*w*/*w*) prior to crushing: 0% (control) and 3% (fortified). The choice of the percentage of olive leaves was based on previous reports [32,33]. According the above studies, 3% was the optimal percentage of olive leaves which can be added to improve oil quality without any negative effects. Only healthy fruits without any kind of infection or physical damage were processed. After harvesting, fresh olives (2.5–3.0 kg) were washed and crushed with a hammer crusher, and the paste, mixed at 25 °C for 30 min, was centrifuged without the addition of warm water. The oil yield from each extraction was 200–250 mL/kg. The oil produced was then transferred into dark glass bottles and stored in the dark at 4 °C until analysis.

### 3.2. Methods

#### 3.2.1. Quality Parameters

Determination of physicochemical quality parameters (free acidity and peroxide values) was carried out following the analytical methods described by Regulation EEC/2568/91 and EEC/1429/92 of the European Union Commission (European Union Commission Regulation, 1991, 1992).

Free acidity, given as percentage of oleic acid, was determined by titration of the oil dissolved in an ethanol–ether solution (1:1, *v*/*v*) with a 0.1 M potassium hydroxide ethanolic solution. The peroxide value, expressed in milliequivalents of active oxygen per kg oil (meq/kg), was determined as follows: a mixture of oil and chloroform–acetic acid was left to react with a solution of potassium iodide (10.5 M) in the darkness. The free iodine was then titrated with a sodium thiosulfate solution (0.01 N).

#### 3.2.2. Pigment Concentration

##### Oil Pigment Determination

One millilitre of oil was diluted ten-fold in *n*-hexane. Chlorophylls and carotenoids were determined colourimetrically as previously described [55]. The maximum absorption at 670 nm was related to the chlorophyll fraction and that at 470 nm to the carotenoid one. The specific extinction coefficients considered for calculation were 613 for pheophytin, as a major component of the chlorophyll fraction, and 2000 for lutein, as a major component of the carotenoid fraction. The pigment concentrations were calculated as follows:

Chlorophyll (mg/kg) = (A_670_ × 10^6^)/(613 × 100 × d)

Carotenoids (mg/kg) = (A_470_ × 10^6^)/(2000 × 100 × d)

where A is the absorbance and d is the spectrophotometer cell thickness (1 cm).

##### Leaf Pigment Determination

Fresh leaf tissue (0.1 g) was ground in a mortar with sand and 70% ethanol solution. The homogenates were then filtered and washed with 70% ethanol (up to 5 mL). After centrifugation for 10 min at 12,100× *g*, absorbance was read at 646.6 and 663.6 nm for chlorophylls and at 480 nm for carotenoids. Concentrations of total chlorophylls and total carotenoids (µg/g DW) were calculated according to Porra et al. [56].

### 3.3. Extraction of Phenolic Compounds

#### 3.3.1. Fresh Leaves

Leaf samples (0.2 g) were ground in a mortar at room temperature with 70% methanol containing 1% HCl. The homogenates were sonicated for 30 min and centrifuged at 12,100× *g* for 30 min at 4 °C. The supernatants were stored at −20 °C and used to determine both phenolic compounds and antioxidant activity.

#### 3.3.2. Olive Oils

Phenolic compounds of olive oils were extracted according to Rotondi et al. [57]. Two grams of oil were added to 1 mL of *n*-hexane and 2 mL of a methanol/water (70:30, *v*/*v*) solution in a 10 mL centrifuge tube. After vigorous mixing, tubes were centrifuged for 10 min. The hydroalcoholic phase was collected, and the hexane phase was re-extracted twice with 2 mL of a methanol/water (70:30, *v*/*v*) solution. The hydroalcoholic fractions were combined, washed with 2 mL of *n*-hexane to remove the residual oil and vacuum-dried.

### 3.4. Total Polyphenol and Flavonoid Concentrations

Total phenolic content was estimated by the Folin Ciocalteu method as described by Singleton and Rossi [58]. To the extract, diluted with distilled water, 1 mL of sodium carbonate (20%) and 1 mL of Folin Ciocalteu reagent were added. The mixture was allowed to stand in a water bath for 30 min at 40 °C. The concentration of the total phenolic compounds was expressed as mg of gallic acid equivalents. The absorbance was measured at 765 nm using a UV–vis spectrophotometer (VARIAN, Milan, Italy). The experiments were performed in triplicate, and mean values and standard deviations were calculated using the Microsoft Excel software (Microsoft Corporation, Redmond, WA, USA).

The total flavonoid concentration was determined by the aluminium trichloride method using catechin as reference compound [59]. A 5% NaNO_2_ solution was added to the extract, followed after 6 min by 10% aluminium trichloride. The mixture was incubated for further 5 min and then 1 M NaOH was added. The final volume was 2.5 mL. After 15 min of incubation, the absorbance at 510 nm was detected. Total flavonoid concentration was expressed as mg of catechin equivalents.

### 3.5. Free-Radical Scavenging Ability

The free-radical scavenging activity of samples was determined by the ABTS radical cation decolourisation assay described by Pellegrini et al. [60]. The radical solution was generated by adding 7 mM ABTS solution to 4.9 mM potassium persulfate. Before use, the radical solution was diluted with ethanol to obtain an absorbance of 0.700 at 734 nm. A control containing ethanol and ABTS^•+^ solution was also prepared, and the absorbance was taken as the initial. After a 15 min incubation period at room temperature, the final absorbance was read at 734 nm. Calculations were performed by percentage of inhibition of the ABTS cation radical as follows:

% of inhibition = ((initial Abs − final Abs)/initial Abs) × 100

To quantify antioxidant capacity, a calibration curve of the percentage of inhibition against Trolox in the range 2–20 nmol was used.

### 3.6. HPLC Analysis of Phenolic Compounds

Qualitative and quantitative analysis were performed by reverse-phase HPLC (RP-HPLC) [61]. Twenty microlitres of extract were injected into a Waters model 515 HPLC system fitted with a 3.9 mm × 150 mm Nova-Pak C18 column (Waters, Milford, MA, USA). Detection was conducted at 280 nm using a Waters 2487 dual λ UV–visible detector. Mobile phase A contained 98% water and 2% acetic acid, and mobile phase B contained 68% water, 30% acetonitrile and 2% acetic acid. A linear gradient of 10 to 95% mobile phase B was run for 90 min at 1 mL/min. The identity of the phenolic acids was confirmed by cochromatography on HPLC with authentic standards (Sigma Chemical Co., St. Louis, MO, USA), and quantification was performed using a standard curve in the range of 20 to 200 ng of standard mixtures containing gallic, protocatechuic, *p*-hydroxybenzoic, chorogenic, vanillic, caffeic, syringic, *p*-coumaric, ferulic, tyrosol, hydroxytyrosol, vanillin and oleuropein. Chromatogram analysis was performed by the software Millennium 32 (Waters).

### 3.7. Extraction and Detection of Tocopherols (Vitamin E)

Tocopherols were determined in the lipid extracts from olive leaves and in oils. Extractions were performed in the dark as previously reported [54] and according to the method of Gimeno et al. [62]. Tocopherol isoforms (α and γ) were determined by isocratic RP-HPLC using a Shimadzu apparatus (model LC-20AD) with an electrochemical detector (Metrohm model 791, Varese, Italy) equipped with a glassy carbon electrode and LC Solution software (Shimadzu) for the integration of peaks. Detection was performed according to Galatro et al. [63] at +0.6 V at 25 °C with a Nova Pak C-18 4 μm column (3.9 × 150 mm). The extracts were eluted with 95% methanol containing 20 mM LiClO_4_ at a flow rate of 1 mL min^−1^. For identification and quantification of peaks, a calibration curve was prepared using standard mixtures of α-, β-, γ- and δ- tocopherol provided by Sigma (Milan, Italy) in the range of 25 to 75 ng.

### 3.8. Statistical Analysis

The results are means from three replicates. All data are reported as mean values ± SE. The significance of differences among mean values was determined by one-way ANOVA. Comparisons among means were performed using Duncan’s multiple range test. Means in tables and figures accompanied by different letters are significantly different at *p* ≤ 0.05.

## 4. Conclusions

The present study confirms the dependence of olive oil quality on the cultivar, besides geographical location, climate and soil characteristics. The Neb Jmel oil showed the best chemical composition with the lowest free acidity and peroxide values, the highest chlorophyll, total phenol and total flavonoid concentrations as well as antioxidant activity. The addition of olive leaves (3%) to Neb Jmel and Oueslati oils affected both their quality and chemical composition, mainly conferring an increased resistance to oxidation as well as improving the nutritional qualities. In particular, a remarkable increase in oleuropein derivatives was observed, which was responsible for the enhancement in total phenolic amounts. As Oueslati leaves are particularly enriched in chlorophyll, carotenoids, flavonoids and oleouperin derivatives, their addition during oil extraction may have prevented the oxidation and the formation of peroxides. Thus, in the Oueslati variety leaf addition reduced both peroxide values and free acidity of oils, allowing them to be still classified as extra virgin olive oils. The enrichment of the oils with antioxidant compounds from the leaves also led to a remarkable increase in the nutritional quality of the Oueslati oil, which became similar to the Neb Jmel one. In conclusion, the addition of a small percentage of olive leaves could improve the nutraceutical properties of extra virgin olive oil by increasing the phenolic compound content. These compounds, together with tocopherols, play a protective role against oxidative stress, being also able to extend the extra virgin olive oil shelf-life due to their antioxidative properties.

## Figures and Tables

**Table 1:** Quality parameters and chlorophyll and carotenoid contents of Neb Jmel and Oueslati olive oils extracted with and without addition of olive leaves (3%).

Parameter	Neb Jmel		Oueslati	
	Oil	Oil + Leaves	Oil	Oil + Leaves
Free acidity (%C18:1)	0.56 ± 0.05 ^b^	0.57 ± 0.03 ^b^	1.00 ± 0.00 ^a^	0.60 ± 0.01 ^b^
Peroxide value (meq O_2_/kg)	6.00 ± 0.71 ^c^	6.00 ± 0.50 ^c^	34.00 ± 9.53 ^a^	15.33 ± 3.21 ^b^
Chlorophyll (mg/kg)	8.37 ± 1.32 ^b^	14.91 ± 2.91 ^a^	5.33 ± 1.22 ^c^	16.51 ± 2.95 ^a^
Carotenoids (mg/kg)	2.95 ± 0.13 ^c^	5.21 ± 0.54 ^b^	2.76 ± 0.60 ^c^	7.26 ± 0.61 ^a^

Data are means of three independent experiments ± SE (*n* = 3). Means followed by different letters are significantly different at *p* ≤ 0.05 as determined by Duncan’s multiple range test. SE, standard error; meq, milliequivalent.

**Table 2:** Chlorophyll, carotenoid and total phenols, flavonoids and tocopherol (α and γ) contents, and ABTS radical cation (ABTS^•+^) scavenging activity of Neb Jmel and Oueslati olive leaves.

Parameter	Neb Jmel	Oueslati
Chlorophyll (µg/g DW)	506.08 ± 10.22 ^b^	829.29 ± 80.06 ^a^
Carotenoids (µg/g DW)	26.90 ± 4.45 ^b^	44.33 ± 4.38 ^a^
Total phenolics (mg GA eq/g DW)	62.84 ± 1.29 ^b^	67.91 ± 3.18 ^a^
Total flavonoids (mg CA eq/g DW)	5.85 ± 1.16 ^b^	13.61 ± 0.81 ^a^
ABTS^•+^ (µmol TE/g DW)	69.05 ± 3.26 ^b^	113.84 ± 5.11 ^a^
γ (µg/g DW)	tr	tr
α (µg/g DW)	82.37 ± 9.95 ^a^	10.12 ± 0.84 ^b^

Data are means of six independent experiments ± SE (*n* = 6). Means followed by different letters are significantly different at *p* ≤ 0.05 as determined by Duncan’s multiple range test. DW, dry weight; GA, gallic acid; CA, catechin; TE, trolox equivalent; α, alpha tocopherol; γ, gamma tocopherol, tr, trace.

**Table 3:** Phenolic compounds (µg/g DW) of Neb Jmel and Oueslati olive leaves.

Parameter	Neb Jmel	Oueslati
	**Secoiridoids**	
Oleuropein derivatives	648.6 ± 92.2 ^b^	1948.6 ± 96.8 ^a^
	**Phenolic Acids**	
Gallic	1.76 ± 0.65 ^a^	0.31 ± 0.25 ^b^
Protocatechuic	37.39 ± 8.68 ^a^	32.39 ± 12.80 ^a^
*p*-Hydroxybenzoic	0.93 ± 0.05 ^b^	4.16 ± 0.41 ^a^
Chlorogenic	1.80 ± 0.00	Tr.
Vanillic	33.84 ± 2.66 ^a^	12.11 ± 1.36 ^b^
Caffeic	1.01 ± 0.05 ^a^	1.19 ± 0.61 ^a^
Syringic	2.28 ± 0.55 ^b^	8.91 ± 2.65 ^a^
Vanillin	2.90 ± 0.11 ^b^	11.32 ± 3.84 ^a^
*p*-Coumaric	5.66 ± 0.52 ^a^	0.87 ± 0.57 ^b^
Ferulic	15.07 ± 0.40 ^b^	37.18 ± 4.71 ^a^
	**Phenolic Alcohols**	
Hydroxytyrosol	5.75 ± 1.40 ^b^	24.03 ± 2.43 ^a^
Tyrosol	3.17 ± 0.76 ^b^	14.46 ± 2.23 ^a^

Data are means of three independent experiments ± SE (*n* = 3). Means followed by different letters are significantly different at *p* ≤ 0.05 as determined by Duncan’s multiple range test.

**Table 4:** Phenolic compounds (mg/kg) of Neb Jmel and Oueslati olive oils extracted with and without addition of olive leaves (3%).

Parameter	Neb Jmel		Oueslati	
	Oil	Oil + Leaves	Oil	Oil + Leaves
	**Secoiridoids**			
**Oleuropein derivatives**	194.4 ± 12.1 ^b^	211.0 ± 1.9 ^a^	126.4 ± 2.6 ^c^	187.6 ± 10.5 ^b^
	**Phenolic Acids**			
**Gallic**	-	-	-	-
**Protocatechuic**	-	-	-	-
***p*-Hydroxybenzoic**	-	-	-	-
**Chlorogenic**	-	-	-	-
**Vanillic**	1.52 ± 0.02 ^a^	1.44 ± 0.05 ^a^	0.77 ± 0.14 ^b^	0.27 ± 0.03 ^c^
**Caffeic**	0.007 ± 0.003 ^b^	0.02 ± 0.0001 ^a^	0.004 ± 0.001 ^b^	0.015 ± 0.0001 ^a^
**Syringic**	0.005 ± 0.004 ^b^	Tr.	0.02 ± 0.01 ^a^	0.02 ± 0.005 ^a^
**Vanillin**	0.15 ± 0.0008 ^a^	0.17 ± 0.01 ^a^	0.17 ± 0.05 ^a^	0.11 ± 0.01 ^a^
***p*-Coumaric**	0.11 ± 0.008 ^b^	0.12 ± 0.0004 ^b^	0.29 ± 0.01 ^a^	0.32 ± 0.04 ^a^
**Ferulic**	0.10 ± 0.005 ^a^	0.11 ± 0.005 ^a^	0.11 ± 0.01 ^a^	0.17 ± 0.01 ^a^
		**Phenolic Alcohols**		
**Tyrosol**	17.97 ± 0.018 ^a^	14.67 ± 0.59 ^b^	1.57 ± 0.05 ^c^	1.07 ± 0.13 ^c^
**Hydroxytyrosol**	3.57 ± 0.02 ^b^	4.13 ± 0.11 ^a^	1.38 ± 0.20 ^c^	1.17 ± 0.12 ^c^

Data are means of three independent experiments ± SE (*n* = 3). Means followed by different letters are significantly different at *p* ≤ 0.05 as determined by Duncan’s multiple range test.

## References

[1] Boudhrioua N., Bahloul N., Ben Slimen I., Kechaou N. (2009). Comparison on the total phenol concentrations and the color of fresh and infrared dried olive leaves. Ind. Crops Prod..

[2] Zinnai A., Venturi F., Andrich L., Silvestri S., Andrich G. (2014). A kinetic method to evaluate the effect of environmental variability on the quality of an extra virgin olive oil. Agrochimica.

[3] Bouaziz M., Chamkha M., Sayadi S. (2004). Comparative study on phenolic concentration and antioxidant activity during maturation of the olive cultivar Chemlali from Tunisia. J. Agric. Food Chem..

[4] Harwood J.L., Yaqoob P. (2002). Nutritional and health aspects of olive oil. Eur. J. Lipid Sci. Technol..

[5] Turner R., Etiene N., Garcia-Alonso M., de Pascual-Teresa S., Minihane A.M., Weinberg P.D., Rimbach G. (2010). Antioxidant and anti-atherogenic activities of olive oil phenolics. Int. J. Vitaminol. Nutr. Res..

[6] Boss A., Bishop K.S., Marlow G., Barnett M.P.G., Ferguson L.R. (2016). Evidence to Support the Anti-Cancer Effect of Olive Leaf Extract and Future Directions. Nutrients.

[7] Zinnai A., Venturi F., Quartacci M.F., Sanmartin C., Favati F., Andrich G. (2016). Solid carbon dioxide to promote the extraction of extra-virgin olive oil. Grasas y Aceites.

[8] Zhang X., Cao J., Zhong L. (2009). Hydroxytyrosol inhibits pro-inflammatory cytokines, iNOS, and COX-2 expression in human monocytic cells. Naunyn Schmiedebergs Arch. Pharmacol..

[9] Venturi F., Sanmartin C., Taglieri I., Nari A., Andrich G., Terzuoli E., Donnini S., Nicolella C., Zinnai A. (2017). Development of Phenol-Enriched Olive Oil with Phenolic Compounds Extracted from Wastewater Produced by Physical Refining. Nutrients.

[10] Bayram B., Ozcelik B., Grimm S., Roeder T., Schrader C., Ernst I.M., Wagner A.E., Grune T., Frank J., Rimbach G. (2012). A diet rich in olive oil phenolics reduces oxidative stress in the heart of SAMP8 mice by induction of Nrf 2-dependent gene expression. Rejuvenation Res..

[11] Gill C.I.R., Boyd A., McDermott E., McCann M., Servili M., Selvaggini R., Taticchi A., Esposto S., Montedoro G., McGlynn H. (2005). Potential anti-cancer effects of virgin olive oil phenols on colorectal carcinogenesis models in vitro. Int. J. Cancer.

[12] Rigacci S., Guidotti V., Bucciantina M., Parri M., Nediani C., Cerbai E., Stefani M., Berti A. (2010). Oleuropein aglycon prevents cytotoxic amyloid aggregation of human amylin. J. Nutr. Biochem..

[13] Schaffer S., Podstawa M., Visioli F., Bogani P., Muller W.E., Eckert G.P. (2007). Hydroxytyrosol-rich olive mill wastewater extract protects brain cells in vitro and ex vivo. J. Agric. Food Chem..

[14] Beltran G., Aguilera M.P., Del Rio C., Sanchez S., Martinez L. (2005). Influence of fruit ripening process on the natural antioxidant concentration of Hojiblanca virgin olive oils. Food Chem..

[15] Gambacorta G., Faccia M., Previtali M.A., Pati S., La Notte E., Bioano A. (2010). Effects of olive maturation and stoning on quality indices and antioxidant concentration of extra virgin oils (cv. *Coratina*) during storage. J. Food Sci..

[16] Cinquanta L., Esti M., Notte E. (1997). Evaluation of phenolic compounds in virgin olive oil during storage. J. Am. Oil Chem. Soc..

[17] Malheiro R., Casal S., Teixeira H., Bento A., Pereira J.A. (2013). Effect of Olive Leaves Addition during the Extraction Process of Overmature Fruits on Olive Oil Quality. Food Bioprocess Technol..

[18] Bouaziz M., Fki I., Jemai H., Ayadi M., Sayadi S. (2008). Effect of storage on refined and husk olive oils composition: Stabilization by addition of natural antioxidants from Chemlali olive leaves. Food Chem..

[19] Bubonja-Sonje M., Giacometti J., Abram M. (2011). Antioxidant and antilisterial activity of olive oil, cocoa and rosemary extract polyphenols. Food Chem..

[20] Kiritsakis K., Kontominas M.G., Kontogiorgis C., Litina D.H., Moustakas A., Kiritsakis A. (2010). Composition and Antioxidant Activity of Olive Leaf Extracts from Greek Olive Cultivars. J. Am. Oil Chem. Soc..

[21] Paiva-Martins F., Correia R., Félix S., Ferreira P., Gordon M.H. (2007). Effects of enrichment of refined olive oil with phenolic compounds from olive leaves. J. Agric. Food Chem..

[22] IOC (2009). Norme Commercial Applicable Aux Huiles D’olive Et Aux Huiles De Grignon D’olive.

[23] Gutierrez F., Varona I., Albi M.A. (2000). Relation of acidity and sensory quality with sterol concentration of olive oil from stored fruit. J. Agric. Food Chem..

[24] Ben Mansour A., Gargouri B., Flamini G., Bouaziz M. (2015). Effect of Agricultural Sites on Differentiation between Chemlali and Neb Jmel Olive Oils. J. Oleo Sci..

[25] Ouni Y., Flamini G., Issaoui M., Ben Youssef N., Luigi C.P., Hammami M., Daoud D., Mokhta Z. (2011). Volatile compounds and compositional quality of virgin olive oil from Oueslati variety: Influence of geographical origin. Food Chem..

[26] Martinéz-Suárez J.M. (1973). Recientes estúdios de la almazara experimental del instituto de la grasa. Rivista Italiana delle Sostanze Grasse.

[27] Matthaüs B., Guillaume D., Gharby S., Haddad A., Harhar H., Charrouf Z. (2010). Effect of processing on the quality of edible argan oil. Food Chem..

[28] Gharby S., Harhar H., Guillaume D., Haddad A., Matthäus B., Charrouf Z. (2011). Oxidative Stability of Edible Argan Oil: A TwoYear Period Study. LWT Food Sci. Technol..

[29] Gutiérrez F., Perdiguero S., Gutiérrez R., Olías J.M. (1992). Evaluation of the bitter taste in virgin olive oil. J. Am. Oil Chem. Soc..

[30] Sgherri C., Perez-Lopez U., Pinzino C., Baily J.R. (2015). Antioxidant properties of food products containing lycopene are increased by the presence of chlorophyll. Lycopene: Food Sources, Potential Role in Human Health and Antioxidant Effects.

[31] Abaza L., Taamalli W., Ben Temime S., Daoud D., Gutierrez F., Zarrouk M. (2005). Natural antioxidant composition as correlated to stability of some Tunisian virgin olive oils. Rivista Italiano delle Sostanze Gresse.

[32] Giovacchino L.D., Angerosa F., Giacinto L.D. (1996). Effect of mixing leaves with olives on organoleptic quality of oil obtained by centrifugation. J. Am. Oil Chem. Soc..

[33] Sevim D., Tuncay O. (2013). Effect of Olive Leaves Addition before Extraction of Turkish Olive Cultivars on Olive Oil Minor Components and Antioxidant Activity. Open Access Sci. Rep..

[34] Kandaswami C., Lee L.T., Lee P.P., Hwang J.J., Ke F.C., Huang Y.T., Lee M.T. (2005). The antitumor activities of flavonoids. In Vivo.

[35] Crozier A., Ashihara H. (2006). Plant Secondary Metabolites and the Human Diet.

[36] Ebrahimi N., Khanikia G.J., Keshavarz S.A., Shariatifar N., Nabizadeh R., Sadighara P. (2015). Spectrophotometric method for quantification of flavonoids in olive oil supplied from Tehran market of Iran. J. Food Saf. Hyg..

[37] Gorinstein S., Martin-Belloso O., Katrich E., Lojek A., Cíz M., Gligelmo-Miguel N., Haruenkit R., Park Y.S., Jung S.T., Trakhtenberg S. (2003). Comparison of the concentrations of the main biochemical compounds and the antioxidant activity of some Spanish olive oils as determined by four different radical scavenging tests. J. Nutr. Biochem..

[38] Samaniego S.C., Troncoso González A.M., García-Parrilla M.C., Quesada Granados J.J., López García de la Serrana H., López Martínez M.C. (2007). Different radical scavenging tests in virgin olive oil and their relation to the total phenol content. Anal. Chim. Acta.

[39] Ben Salah M., Abdelmelek H., Abderraba M. (2012). Study of Phenolic Composition and Biological Activities Assessment of Olive Leaves from different Varieties Grown in Tunisia. J. Med. Chem..

[40] Chin K.Y., Ima-Nirwana S. (2018). The Role of Vitamin E in Preventing and Treating Osteoarthritis—A Review of the Current Evidence. Front. Pharmacol..

[41] Sedef N., Karakaya S. (2009). Olive tree (*Olea europaea*) leaves: Potential beneficial effects on human health. Nutr. Rev..

[42] Cortesi N., Rovellini P., Fusari P. (2002). Dosaggio dei biofenoli degli oli vergini di oliva: Idrossitirosolo, tirosolo, agliconi secoiridoidi, acidi secoridoidi, lignani e flavonoidi. Rivista Italiana delle Sostanze Grasse.

[43] De Leonardis A., Macciola V., Lembo G., Aretini A., Nag A. (2007). Studies on oxidative stabilisation of lard by natural antioxidants recovered from olive-oil mill wastewater. Food Chem..

[44] Khoufi S., Aloui F., Sayadi S. (2008). Extraction of antioxidants from olive mill wastewater and electro-coagulation of exhausted fraction to reduce its toxicity on anaerobic digestion. J. Hazard. Mater..

[45] El-Abbassi A., Kiai H., Hafidi A. (2012). Phenolic profile and antioxidant activities of olive mill wastewater. Food Chem..

[46] Azaizeh H., Halahlih F., Najami N., Brunner D., Faulstich M., Tafesh A. (2012). Antioxidant activity of phenolic fractions in olive mill wastewater. Food Chem..

[47] Kelebek H., Selli S., Kol O. (2017). Quantitative determination of phenolic compounds using LC-DAD-ESI-MS/MS in cv. Ayvalik olive oils as affected by harvest time. J. Food Meas. Charact..

[48] Lucas A., Martinez E., Rincón J., Blanco M.A., Garcia I. (2002). Supercritical fluid extraction of tocopherol concentrates from olive tree leaves. J. Supercrit. Fluids.

[49] Franco M.N., Galeano-Diaz T., Sanchez J., De Miguel C., Martin-Vertedor D. (2014). Total phenolic compounds and tocopherols profiles of seven olive oil varieties grown in the south-west of Spain. J. Oleo Sci..

[50] Cunha S.S., Fernandes J.O., Oliveira M.B. (2006). Quantification of free and esterified sterols in Portuguese olive oils by solid-phase extraction and gas chromatography-mass spectrometry. J. Chromatogr. A.

[51] Baccouri O., Guerfel M., Baccouri B., Cerretani L., Bendini A., Lercker G., Zarrouk M., Daoud Ben Miled D. (2008). Chemical composition and oxidative stability of Tunisian monovarietal virgin olive oils with regard to fruit ripening. Food Chem..

[52] Krichene D., Taamalli W., Daoud D., Salvador M.D., Fregapane G., Zarrouk M. (2007). Phenolic Compounds, Tocopherols and others minor components in virgin olive oils of some Tunisian Varieties. J. Food Biochem..

[53] Manai-Djebali H., Krichène D., Ouni Y., Gallardo L., Sánchez J., Osorio E., Daoud D., Guido F., Zarrouk M. (2012). Chemical profiles of five minor olive oil varieties grown in central Tunisia. J. Food Compos. Anal..

[54] Sgherri C., Kadlecova Z., Prdossi A., Navari-Izzo F., Izzo R. (2008). Irrigation with Diluted Seawater Improves the Nutritional Value of Cherry Tomatoes. J. Agric. Food Chem..

[55] Mínguez-Mosquera M.I., Rejano-Navarro L., Gandul-Rojas B., Sánchez-Gómez A.H., Garrido-Fernández J. (1991). Color–Pigment Correlation in Virgin Olive Oil. J. Am. Oil Chem. Soc..

[56] Porra R.J., Thompson W.A., Kriedemann P.E. (1989). Determination of accurate extinction coefficients and simultaneous equations for assaying chlorophylls a and b extracted with four different solvents: Verification of the concentration of chlorophyll standards by atomic absorption spectrometry. Biochim. Biophys. Acta.

[57] Rotondi A., Bendini A., Cerretani L., Mari M., Lercker G., Gallina-Toschi G.T. (2004). Effect of Olive Ripening Degree on the Oxidative Stability and Organoleptic Properties of Cv. Nostrana di Brisighella Extra Virgin Olive Oil. J. Agric. Food Chem..

[58] Singleton V.L., Rossi J.A. (1965). Colorimentry of total phenolics with phosphomolybdic phosphotungstic acid reagents. Am. J. Enol. Vitic..

[59] Zhishen J., Mengcheng T., Jianming W. (1999). The determination of flavonoid contents in mulberry and their scavenging effects on superoxide radicals. Food Chem..

[60] Pellegrini N., Re R., Yang M., Rice-Evans C. (1999). Screening of dietary carotenoids and carotenoid-rich fruit extracts for antioxidant activities applying 2,2′-azinobis (3-ethylbenzothiazolyne-6-sulfonic acid) radical cation decolorization assay. Methods Enzymol..

[61] Talcott S.T., Howard L.R. (1999). Phenolic autoxidation is responsible for color degradation in processed carrot puree. J. Agric. Food Chem..

[62] Gimeno E., Castellote A.I., Lamuela-Raventós R.M., de la Torre M.C., López-Sabater M.C. (2000). Rapid determination of vitamin E in vegetable oils by reversed-phase high-performance liquid chromatography. J. Chromatogr. A.

[63] Galatro A., Simontacchi M., Puntarulo S. (2001). Free radical generation and antioxidant content in chloroplasts from soybean leaves exposed to ultraviolet-B. Physiol. Plant..

